# The identification of a RNA splice variant in *TULP1* in two siblings with early‐onset photoreceptor dystrophy

**DOI:** 10.1002/mgg3.660

**Published:** 2019-04-04

**Authors:** Sanne K. Verbakel, Zeinab Fadaie, B. Jeroen Klevering, Maria M. van Genderen, Ilse Feenstra, Frans P. M. Cremers, Carel B. Hoyng, Susanne Roosing

**Affiliations:** ^1^ Department of Ophthalmology Donders Institute for Brain Cognition and Behavior Radboud University Medical Center Nijmegen The Netherlands; ^2^ Department of Human Genetics Donders Institute for Brain Cognition and Behavior Radboud University Medical Center Nijmegen The Netherlands; ^3^ Bartiméus Diagnostic Center for Complex Visual Disorders Zeist The Netherlands; ^4^ Department of Ophthalmology University Medical Center Utrecht Utrecht The Netherlands

**Keywords:** early‐onset retinal dystrophy, intronic variant, TULP1, whole exome sequencing

## Abstract

**Background:**

Early‐onset photoreceptor dystrophies are a major cause of irreversible visual impairment in children and young adults. This clinically heterogeneous group of disorders can be caused by mutations in many genes. Nevertheless, to date, 30%–40% of cases remain genetically unexplained. In view of expanding therapeutic options, it is essential to obtain a molecular diagnosis in these patients as well. In this study, we aimed to identify the genetic cause in two siblings with genetically unexplained retinal disease.

**Methods:**

Whole exome sequencing was performed to identify the causative variants in two siblings in whom a single pathogenic variant in *TULP1* was found previously. Patients were clinically evaluated, including assessment of the medical history, slit‐lamp biomicroscopy, and ophthalmoscopy. In addition, a functional analysis of the putative splice variant in *TULP1* was performed using a midigene assay.

**Results:**

Clinical assessment showed a typical early‐onset photoreceptor dystrophy in both the patients. Whole exome sequencing identified two pathogenic variants in *TULP1*, a c.1445G>A (p.(Arg482Gln)) missense mutation and an intronic c.718+23G>A variant. Segregation analysis confirmed that both siblings were compound heterozygous for the *TULP1* c.718+23G>A and c.1445G>A variants, while the unaffected parents were heterozygous. The midigene assay for the c.718+23G>A variant confirmed an elongation of exon 7 leading to a frameshift.

**Conclusion:**

Here, we report the first near‐exon RNA splice variant that is not present in a consensus splice site sequence in *TULP1*, which was found in a compound heterozygous manner with a previously described pathogenic *TULP1* variant in two patients with an early‐onset photoreceptor dystrophy. We provide proof of pathogenicity for this splice variant by performing an in vitro midigene splice assay, and highlight the importance of analysis of noncoding regions beyond the noncanonical splice sites in patients with inherited retinal diseases.

## 1 INTRODUCTION

Inherited retinal diseases are characterized by the progressive degeneration of photoreceptor and/or retinal pigment epithelium cells, and are a major cause of irrecoverable visual impairment. Retinitis pigmentosa (RP) encompasses the most common group of inherited retinal diseases, with a worldwide prevalence of approximately 1 in 4,000 individuals (Pagon, 1988). RP is characterized by the rod photoreceptor degeneration that precedes cone photoreceptor degeneration. Patients generally present with night blindness, followed by a gradual constriction of the visual field. The visual acuity typically remains relatively preserved until the final stages of disease. Characteristic fundus features include bone spicule pigmentation, attenuation of retinal vessels, and a waxy pallor of the optic disc. RP can follow all Mendelian patterns of inheritance: autosomal dominant, autosomal recessive and X‐linked. In contrast, Leber congenital amaurosis (LCA) is considered the most severe form of inherited retinal disease. It is characterized by severe loss of visual function, nystagmus, photophobia, amaurotic pupils (i.e., sluggish or near‐absent pupillary responses), high hyperopia, oculo‐digital signs such as poking, pressing, and rubbing the eyes, and severely reduced or absence of electroretinogram responses. LCA is generally inherited in an autosomal recessive manner (den Hollander, Roepman, Koenekoop, & Cremers, 2008; Weleber, Francis, Trzupek, & Beattie, 1993).

Early‐onset RP and LCA represent a continuum of retinal dystrophies, and are generally differentiated based on the age of onset; patients with an onset after infancy (variably defined as age one or two) are diagnosed as having RP, while LCA generally presents in the first months of life (Kumaran, Moore, Weleber, & Michaelides, 2017). This arbitrary cut‐off point gives rise to large clinical and genetic overlap between both phenotypes. In addition, both RP and LCA display large clinical and genetic heterogeneity. The genetic heterogeneity is illustrated by the 87 genes that have been associated with nonsyndromic RP, and the 25 genes that are associated with LCA (Retnet; available at https://sph.uth.edu/retnet/) (Kumaran et al., 2017; Verbakel et al., 2018). Ten genes have been associated with both RP and LCA, among which *TULP1* (OMIM: 602280) (Verbakel et al., 2018) .

The *TULP1* gene has been associated with LCA, early‐onset RP and cone(‐rod) dystrophy (Ajmal et al., 2012; den Hollander, van Lith‐Verhoeven et al., 2007; Hanein et al., 2004; Roosing et al., 2013; Ullah et al., 2016). *TULP1* encodes a 542‐aa (61 kDa) photoreceptor‐specific tubby‐like protein (i.e., tubby‐like protein‐1, TULP1) that is likely involved in the transport of several phototransduction proteins from the photoreceptor inner segment to the outer segments, particularly from the opsin (e.g., rhodopsin and cone opsin) and guanylate cyclase carrier pathways (e.g., guanylate cyclase 1 and guanylate cyclase‐activating proteins 1 and 2) (Grossman, Watson, Pauer, Bollinger, & Hagstrom, 2011; Hagstrom, Watson, Pauer, & Grossman, 2012; Xi, Pauer, Marmorstein, Crabb, & Hagstrom, 2005).

To date, a molecular diagnosis can be identified by whole exome sequencing (WES) in approximately 60%–70% of RP and LCA patients (Haer‐Wigman et al., 2017; Kumaran et al., 2017; Tiwari et al., 2016; Zhao et al., 2015). In the remaining cases, the causative variants could be located in a gene that has not yet been associated with early‐onset retinal dystrophies. Alternatively, the genetic defect could reside in a gene that has previously been associated with RP, but the mutation may not have been detected using WES because the variant resides in a GC‐rich region, concerns a structural variant, or was not covered for another reason. Finally, the pathogenic variant could have been missed because of too stringent variant filtering procedures or because it resides outside of the coding regions and splice sites. With the advent of therapeutic options for inherited retinal disorders, it becomes essential to also obtain a molecular diagnosis in patients without a conclusive genetic diagnosis.

In recent years, various studies have shown the importance of searching for variants beyond the coding and splice site regions. Up to 15% of LCA patients carry a deep‐intronic mutation (c.2991+1655A>G) in *CEP290* (Coppieters et al., 2010; den Hollander et al., 2006; Perrault et al., 2007) and these patients may benefit from an upcoming treatment with antisense oligonucleotides (AONs) (Dulla et al., 2018)(ClinicalTrials.gov NCT03140969). Additionally, studies in Stargardt disease have identified numerous variants leading to an alternative splicing or pseudoexon inclusion in *ABCA4* (Albert et al., 2018). In this study, we provide evidence for pathogenicity of the first intronic variant outside of the splice site consensus sequence in *TULP1*, which we coin a near‐exon aberrant RNA (NEAR) splice variant, segregating with a previously described pathogenic missense variant in two siblings with early‐onset retinal dystrophy.

## 2 METHODS

### 2.1 Ethical compliance

The study adhered to the tenets of the Declaration of Helsinki and was approved by the local ethics committee. Written informed consent was obtained from both patients and their parents prior to inclusion in this study.

### 2.2 Clinical evaluation

A family with two siblings with an autosomal recessive early‐onset retinal dystrophy was clinically examined at the Radboud university medical center in Nijmegen, the Netherlands. Clinical data were obtained from the medical records of the patients. Patients’ medical history was registered with special attention for the age at onset, initial symptoms and the course of the disease. In addition, both patients were re‐evaluated after the identification of the genetic cause of disease. We performed a detailed ophthalmic examination, which included best‐corrected visual acuity, slit‐lamp biomicroscopy, and ophthalmoscopy. Fundus photography, spectral‐domain optical coherence tomography (SD‐OCT; Spectralis HRA+OCT, Heidelberg Engineering, Heidelberg, Germany), and fundus autofluorescence (FAF; HRA+OCT, Heidelberg Engineering, Heidelberg, Germany) imaging were performed. The visual field was assessed using a Goldmann perimeter. Full‐field electroretinography (ffERG) recordings were performed according to the International Society for Clinical Electrophysiology of Vision (ISCEV) guidelines and assessed applying local standard values (McCulloch et al., 2015).

### 2.3 Genetic analysis

Genomic DNA was extracted from peripheral lymphocytes according to standard procedures. WES was performed in a certified DNA diagnostic laboratory in both siblings (Haer‐Wigman et al., 2017). The exome was enriched using Agilent's SureSelectXT Human all Exon V5 (Agilent Technologies, Santa Clara, CA). Subsequently, next‐generation sequencing using an Illumina HiSeq 4000 sequencer (Illumina, Inc. San Diego, CA), read alignment to the human reference genome (Genome Reference Consortium Human Reference 37/hg19) using Burrows‐Wheeler Aligner, and variant calling with the Genome Analysis Toolkit were performed at BGI‐Europe (Copenhagen, Denmark). Copy number variants were detected using CoNIFER 0.2.0, and variants were annotated using a custom designed in‐house annotation strategy.

### 2.4 Variant prioritizing

Prioritizing candidate variants for causality was based on their presence in both affected siblings, a minor allele frequency of < 0.5% in ExAC, dbSNP and the Nijmegen in‐house database consisting of 15,576 individuals, their effect (i.e., nonsense, frameshift, canonical (donor +1 and +2, acceptor −1 and −2) and noncanonical (donor +3 to +6, acceptor −3 to −14) splice site variants), and the occurrence in a homozygous or compound heterozygous state. Moreover, for the remaining heterozygous variants in currently known retinal dystrophy‐associated genes, we manually assessed the BAM‐files to verify if all exons (potentially harboring pathogenic variants) were covered.

The pathogenicity of missense variants was evaluated by combining in silico prediction tools, such as SIFT (http://sift-dna.org/), PolyPhen‐2 (http://genetics.bwh.harvard.edu/pph2/), and Mutation Taster (http://www.mutationtaster.org/), and using the PhyloP score (range −14.1–6.4; predicted pathogenic ≥ 2.7) (Pollard, Hubisz, Rosenbloom, & Siepel, 2010), CADD‐PHRED (range 1–99; predicted pathogenic ≥ 15) (https://cadd.gs.washington.edu/), and Grantham scores (range 0–215; predicted pathogenic ≥ 80) (Grantham, 1974). The in silico prediction of noncanonical splice variants was assessed using algorithms (i.e., SpliceSiteFinder‐like (Zhang, 1998), MaxEntScan (http://genes.mit.edu/burgelab/maxent/Xmaxentscan_scoreseq.html), GeneSplicer (https://www.cbcb.umd.edu/software/GeneSplicer/gene_spl.shtml), and Human Splicing Finder (http://www.umd.be/HSF/) embedded in the Alamut Visual software version 2.10 (Interactive Biosoftware, Rouen, France; http://www.interactive-biosoftware.com).

### 2.5 In vitro midigene splice assay

*TULP1* (GenBank: NM_003322.5) is a photoreceptor‐specific protein and not expressed in available somatic cells. Therefore, a functional analysis of the putative splice variant c.718+23G>A in *TULP1* was performed using a midigene assay. We designed two midigene constructs with an insert of 6.3 kb, a wild‐type and mutant multi‐exon splice vector, using a modified protocol of the previously described method (Figure 3).(Sangermano et al., 2016) In short, exon 4–11 of *TULP1* of the genomic DNA from a control individual was amplified using forward primer 5′‐GGAGATCCCTAGGGTGAGGA‐3′ and reverse primer: 5′‐ATCAAAGCGAGAGGCCCTA‐3′. Both primers have an attB1 and attB2 tag at 5′ end to enable Gateway cloning. The wild‐type construct served as a template to generate the mutant construct of c.718+23G>A by mutagenesis PCR. Subsequently, wild‐type and mutant constructs were incorporated into the pCI‐NEO‐*RHO* Gateway‐adapted vector as previously described.(Sangermano et al., 2016) This resulted in a wild‐type midigene c.718+23G and a mutant midigene c.718+23A. Finally, we transfected HEK293T cells with the wild‐type or mutant midigene and studied the transcripts with reverse transcription–polymerase chain reaction with primers in exons 4 and 11 (RT‐PCR) (Forward primer: 5′‐GTCTACGCCAGGTTCCTCAG‐3′ and Reverse primer: 5′‐TCCTCGGGACAGATTGGTAG‐3′).

## 3 RESULTS

### 3.1 Clinical findings

**Figure 1:**
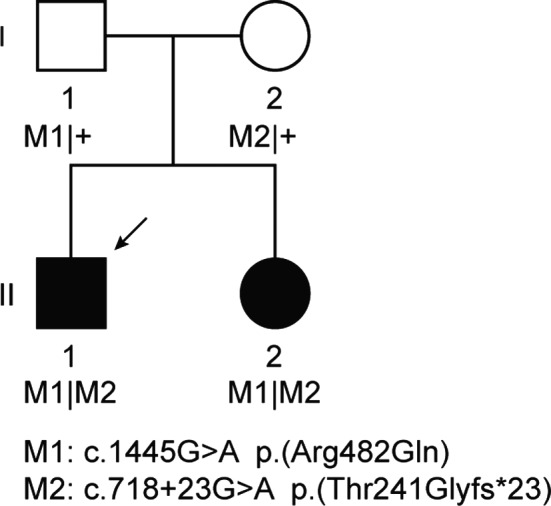
Pedigree of the family included in this study. The variants in *TULP1* segregate with the disease

Figure 1 shows the pedigree of the two siblings we studied, both were from Dutch ancestry and presented with a retinal dystrophy that we classified as very early‐onset forms of RP. An overview of the clinical characteristics at the most recent examination is provided in Table 1. The eldest patient, individual II:1, presented with a fine horizontal nystagmus at the age of three. Subsequently, he developed night blindness that became apparent at the age of five. His visual acuity gradually deteriorated from 20/50 at age five to 20/100 when he was 15 years of age. His younger sister (patient II:2) presented with a subtle horizontal nystagmus at the age of one. Her visual acuity deteriorated from 20/50 at the age of three to 20/60 at age 13, and she also experienced impaired night vision in early childhood. At the most recent examination, both siblings complained of photophobia. In addition, patient II:1 reported photopsias, particularly after a sudden increase in light intensity. Perimetry showed constriction of the visual field from age 11 in patient II:1, with a central island measuring up to 10 degrees at the age of 15 years. In patient II:2, the constriction of the visual field started at age 10 and progressed to a constriction up to 20–30 degrees at the age of 13 years. High hyperopia was present in both siblings, with spherical equivalent refractions ranging from 6.13 to 7.25. Both siblings were in good general health, and no extra‐ocular conditions were reported.

**Table 1:** Clinical features at the most recent examination of the two siblings with *TULP1* pathogenic variants

Patient	Sex	Age (y)	Initial symptom, age (y)	Visual acuity		SER		Lens status	Ophthalmoscopy results	Goldmann perimetry	Electroretinogram, age (y)
				RE	LE	RE	LE				
A‐II:1	M	15	Nystagmus, 3y	20/100	20/200	6,13	7,25	Clear	Sparse bone spicule pigmentation in the periphery, small hyperemic optic discs, and attenuated retinal vessels.	Severely constricted VF to <10°	Scotopic: SR,Photopic: R, 5y
A‐II:2	F	13	Nystagmus, 1y	20/130	20/60	7,00	6,50	Clear	Bone spicule pigmentation in the periphery, small hyperemic optic discs, pigment alterations in the macula, and attenuated retinal vessels.	Constricted VF to 30° (RE) and 20° (LE)	Scoptopic: NR, Photopic: SR, 6y

*Note*. F, female; LE, left eye; M, male; NR, non‐recordable; R, reduced; RE, right eye; SER, spherical equivalent refraction; SR, severely reduced; VF, visual field; y, years.

**Figure 2:**
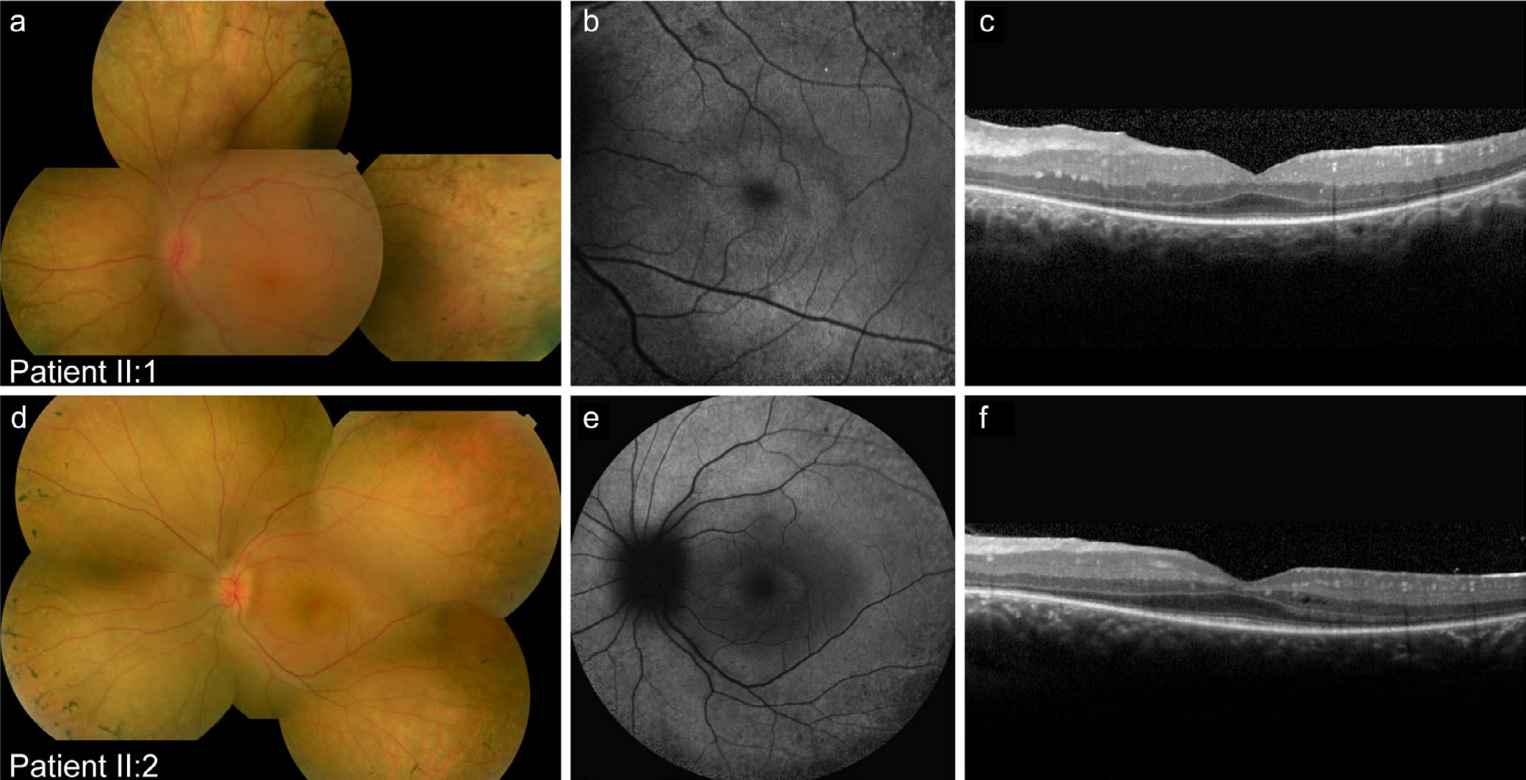
Multimodal images of both siblings. (a–c) Multimodal imaging of the left eye of patient II:1 at the age of 15 years. (a) Composite fundus photograph showing a hyperemic optic disc, sparse bone spicule pigmentation in the periphery, and slightly attenuated vessels. (b) 30° fundus autofluorescence image revealing a characteristic hyperautofluorescent ring, (c) which corresponds to preservation of the ellipsoid zone within the ring, as visible on spectral‐domain optical coherence tomography (SD‐OCT). In addition, SD‐OCT imaging also showed a thickened retina. (d‐f) Multimodal imaging of the left eye of patient II:2 at the age of 13 years. (d) Composite fundus photograph showing a small and hyperemic optic disc, attenuation of the retinal vessels, and bone spicule pigmentation in the periphery. (e) 55° fundus autofluorescence image showing a central hyperautofluorescent ring. (f) The SD‐OCT scan reveals preserved photoreceptor layers at the fovea, and multiple small intraretinal cysts

Ophthalmoscopy showed peripheral bone spicule pigmentation, attenuated retinal vessels, and small hyperemic optic discs (often found in high hyperopia) in both siblings (Figure 2). Fundus autofluorescence imaging revealed the characteristic hyperautofluorescent ring that represents the transition zone between intact and degenerated photoreceptor outer segments. This was confirmed by SD‐OCT images that showed an intact ellipsoid zone layer inside the ring, and loss of the outer retinal layers outside of the hyperautofluorescent ring area. In addition, the SD‐OCT image revealed diffuse thickening of the retina in patient II:1, which proved refractory to treatment with 125 mg oral carbonic anhydrase inhibitors three times a day. Cystoid macular edema was identified in patient II:2 at age 11. However, in her case, the cystoid macular edema revolved after treatment with carbonic anhydrase inhibitors, and did not recur—or at least in a severely reduced fashion—after sustained treatment with 125 mg oral carbonic anhydrase inhibitors two times a day. Electrophysiological examination at the age of five (patient II:1) and six (patient II:2) demonstrated a generalized photoreceptor dystrophy with severely affected photoreceptor responses in a rod‐cone pattern.

### 3.2 Genetic findings

**Figure 3:**
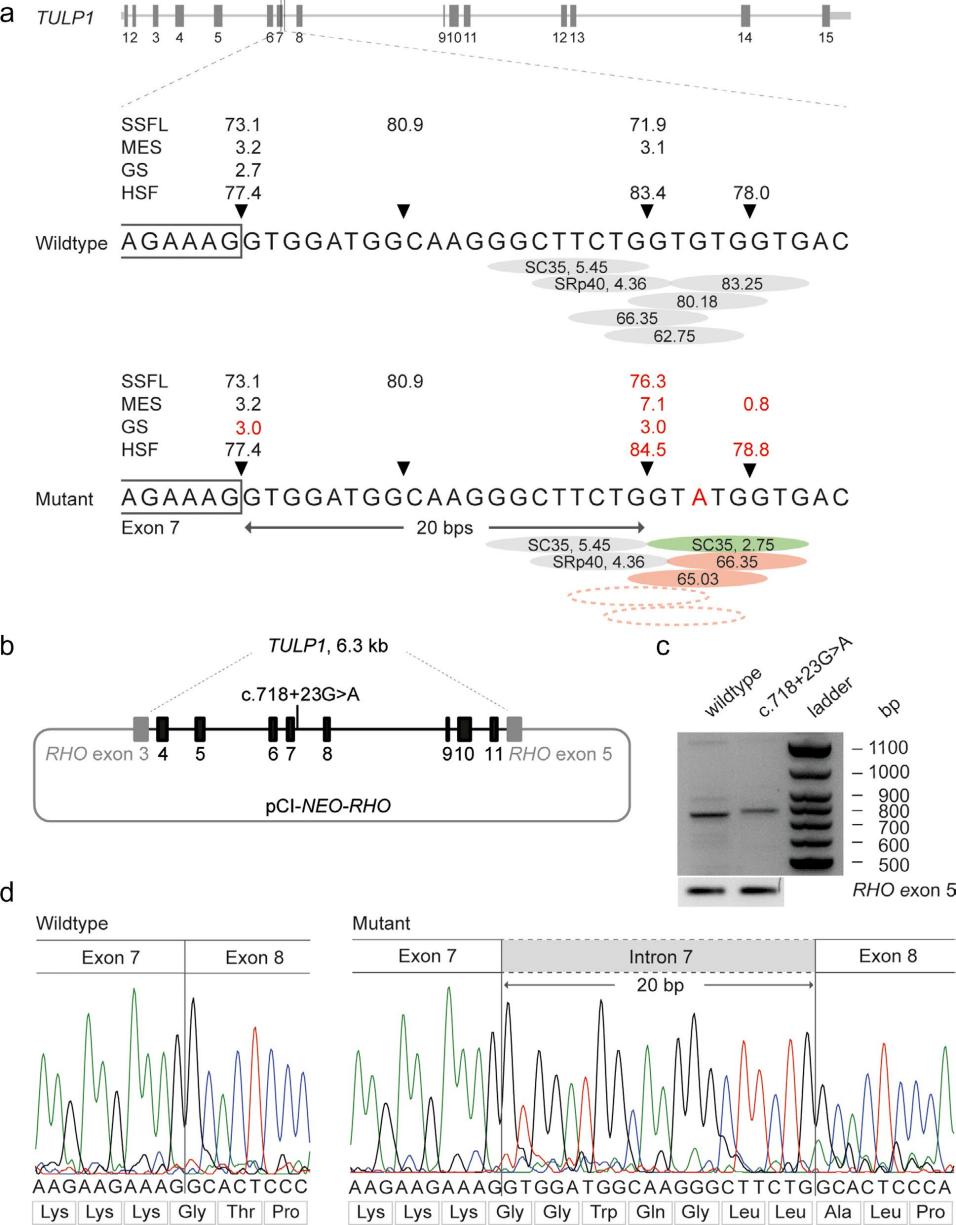
Molecular genetic characterization of the splice effect of the c.718+23G>A variant in *TULP1*. (a) Schematic representation of the *TULP1* gene and enlargement of the wild‐type and mutant DNA sequences at the exon–intron boundary of exon 7 of *TULP1*. The SpliceSiteFinder‐like (SSFL, range 0–100), MaxEntSCan (MES, range 0–12), GeneSplicer (GS, range 0–24) and Human Splicing Finder (HSF, range 0–100) scores for the splice donor site are indicated above the gene. The red “A” highlights the variant c.718+23G>A identified in both siblings. The red numbers represent altered scores compared to the wild‐type. The green circle implies a newly recognized SC35 motif. Dotted circles indicate exonic splice silencers no longer present by prediction tools (b) Schematic representation of the mutant pCI‐NEO‐RHO vector, containing exon 4–11 of the *TULP1* gene used to transfect HEK293T cells with a wild‐type or mutant midigene. (c) RT‐PCR products of the wild‐type and mutant midigene showing the expected 832‐bp wild‐type fragment and a 852‐bp fragment of the mutant midigene corresponding to a 20‐nucleotide elongation of the mRNA encoded by exon 7. The wild‐type fragment was absent in the cells transfected with the mutant midigene. RT‐PCR analysis of *RHO* exon 5 was performed as a control for efficient transfection. (d) Sanger sequence analysis of the RT‐PCR fragments confirmed the wild‐type and the 20‐bp elongation of exon 7 in the mutant

Initial analysis of WES data in patient II:1 detected two heterozygous variants, a c.1445G>A (p.(Arg482Gln)) missense variant in *TULP1* and a c.1567C>T (p.(Arg523*)) nonsense variant in *FAM161A*. Subsequently, WES was performed in patient II:2; she also carried the heterozygous missense variant in *TULP1*, as well as a heterozygous c.3683A>G (p.(Tyr1228Cys)) missense variant in *RPGRIP1*. All coding regions of *TULP1* were covered in the WES data of both siblings, and uncovered exons from *RPGRIP1* and *FAM161A* were Sanger sequenced but did not reveal additional putative pathogenic variants in either sibling. Moreover, no copy number variants were identified in *FAM161A*,* RPGRIP1* or *TULP1* in both individuals. As the *FAM161A* and *RPGRIP1* pathogenic variant were not shared between the siblings, we pursued to study *TULP1* in more detail. The previously described variant c.1445G>A (p.(Arg482Gln) has a CADD‐Phred score of 23.2, a PhyloP score of 6.1, and a Grantham score of 43. (Ajmal et al., 2012) An expanded analysis beyond the coding regions and putative splice site regions resulted in the identification of a heterozygous intronic variant, c.718 + 23G>A (Figure 3), found in both siblings. Segregation analysis confirmed that both siblings were compound heterozygous for the c.718+23G>A and c.1445G>A variants, while the unaffected parents were heterozygous (Figure 1). Although the c.718+23G>A variant, based on splice score prediction algorithms, does not alter the nearby splice donor site, this deep‐intronic variant increased the splice prediction scores at position c.718 + 20 when using programs SpliceSiteFinder‐like (+4.4%), MaxEntScan (+33.33%), GeneSplice (+12.5%), and Human Splicing Finder (+1.1%) (Figure 3). Additionally, c.718+23G>A introduced a new exonic splice enhancer (ESE) motif recognized by the SC35 exonic splice enhancer at the c.718+21 to c.718+29 positions where a putative splice donor site is already located in the wild‐type mRNA, and reduces the number and strength of exonic splice silencer (ESS) motifs present in the reference sequence.

### 3.3 In vitro midigene splice assay

Reverse transcription polymerase chain reaction showed the expected 832‐bp wild‐type fragment (Figure 3). In contrast, the mutant midigene showed a product that corresponds to a larger fragment, and absence of the wild‐type fragment. Sanger sequencing verified that the mutant mRNA product contained a 20‐nucleotide elongation of exon 7, which can be explained by the use of a cryptic splice donor site 20 nucleotides downstream of exon 7 (Figure 3). This elongation causes a frameshift and results in early protein truncation (p.(Thr241Glyfs*23)).

## 4 DISCUSSION

In the present study, we identified a near‐exon aberrant RNA splice variant that we termed a “NEAR” splice variant in *TULP1* in *trans* with a previously described exonic variant in two siblings with early‐onset RP. Both siblings showed a nystagmus, night blindness, and a reduced visual acuity early in life. Fundus examination at the age of 15 (patient II:1) and 13 years (patients II:2) showed bone spicule pigmentation, attenuation of the retinal vessels and a small, hyperemic optic disc. High hyperopia (i.e., refractive error of more than +5.00 D) was present in both siblings. *TULP1*‐associated disease has previously been associated with myopia (den Hollander, Lopez et al., 2007; den Hollander, van Lith‐Verhoeven et al., 2007; Hendriks et al., 2017; Souzeau et al., 2018) and hyperopia (den Hollander, Lopez et al., 2007; Khan, Bergmann, Eisenberger, & Bolz, 2015). However, high hyperopia has only been described in patients with LCA, and is thought to result from impaired emmetropization caused by early‐onset visual impairment (Khan et al., 2015; Weleber et al., 1993).

Both siblings were diagnosed with early‐onset RP based on an onset after the age of one. However, they also show characteristic aspects of LCA such as a nystagmus and hyperopia. The difficulty in classifying such patients arises from the strict and rather arbitrary separation of both entities, when in fact they represent a continuum of retinal dystrophies.

Thus far, three noncanonical splice site variants in the *TULP1* gene (i.e., c.999+5G>C, c.1224A>G, and c.1496‐6C>A) have been reported in patients with *TULP1*‐associated disease (den Hollander, Lopez et al., 2007; Gu et al., 1998; Hagstrom, North, Nishina, Berson, & Dryja, 1998). We identified two pathogenic variants in *TULP1*, a c.1445G>A (p.(Arg482Gln)) missense mutation and an intronic c.718+23G>A variant. The *TULP1* p.(Arg482Gln) mutation, previously described in patients with early‐onset RP, alters the structure and function of the Tubby domain, and is expected to affect TULP1 function (Ajmal et al., 2012). To our knowledge, pathogenicity of mutations in *TULP1* such as the c.718+23G>A variant, which we coin as a NEAR splice variant, has not been described before. Our definition of a NEAR splice variant is a variant located outside of the splice site consensus sequence leading to an alteration of the splicing of a nearby exon, whereas a deep‐intronic variant often leads to the inclusion or alteration of a cryptic exon.

The consequence of a NEAR splice variant depends on the context of the variant, such as the strength of nearby splice acceptor and splice donor sites, the presence and size of flanking exons, and the effect on the appearance or removal of ESE, ESS, intronic splice enhancer, and intronic splice silencer motifs. To assess the effect of this variant on splicing, we generated a midigene assay which contained exon 4 to 11 of *TULP1*. This analysis showed the use of a cryptic splice donor site 20 bp downstream, which causes a shift of the reading frame resulting in early termination of protein synthesis. The severe nature of the c.718+23G>A NEAR splice variant is supported by the absence of a wild‐type fragment in cells transfected with the mutant midigene and corresponds with the severe phenotype observed in the patients.

The donor splice site of exon 7 of the *TULP1* gene contains a fairly weak splice site as indicated by the score of 77.4 for Human Slicing Finder. A natural stronger cryptic splice donor site is present at the c.718+20 position in the wild‐type mRNA with a score of 83.4 for the Human Splicing Finder prediction (Tang, Prosser, & Love, 2016). The presence of ESSs located in and near the c.718+20 position likely explain why the cryptic donor site is not utilized by the splice machinery in wild‐type cells. The c.718+23G>A variant, however, shows a decreased number of ESSs in this region, and consequently enables recognition of the putative splice donor site by the spliceosome (http://www.umd.be/HSF3/). In addition, according to ESE predictions, this variant also creates a new binding site for exonic splice enhancer SC35 at the c.718+21 to c.718+29 positions that strengthens this putative splice donor site and generates preference for the c.718+20 donor instead of the canonical splice donor site. This supports the role of ESEs and ESSs in the splicing process, and highlights the importance of these factors when analyzing the pathogenicity of variants within or outside of the coding and splice site regions.

While this is the first intronic variant outside of the splice site consensus sequences deemed pathogenic in *TULP1*, the causality of deep‐intronic variants has also been described in nine other retinal dystrophy genes: *ABCA4*,* CEP290*,* CHM*,* OA1*,* OAT, OFD1*,* PROM1*,* PRPF31*, and *USH2A* (Bax et al., 2015; Braun et al., 2013; Carss et al., 2017; den Hollander et al., 2006; Liquori et al., 2016; Mayer et al., 2016; Naruto et al., 2015; Rio Frio et al., 2009; Vache et al., 2012; van den Hurk et al., 2003; Webb et al., 2012) (www.dbass.soton.ac.uk). Intronic variants are likely to explain a substantial portion of the current genetically unexplained or monoallelic retinal dystrophy cases, and underscore the importance of genetic tests uncovering those regions, such as whole genome sequencing. Future studies with whole genome sequencing will likely increase the number of genetically solved patients. However, the increase in the use of whole genome sequencing will be accompanied by the detection of a large number of variants of unknown significance, and determining the functional role of these variants will remain a challenge.

The identification of biallelic variants in patients with a retinal dystrophy is essential for eligibility for upcoming genetic therapies. Besides gene augmentation therapy, patients with a *TULP1* NEAR splice variant may benefit from AONs treatment, which can suppress the aberrant splicing effect by binding to the mutated region in the pre‐mRNA. To date, proof‐of‐concept of AONs has been shown in both cell‐based models and animal models for four retinal dystrophy genes: *CEP290* (Collin et al., 2012; Dulla et al., 2018; Garanto et al., 2016; Parfitt et al., 2016), *CHM* (Garanto, van der Velde‐Visser, Cremers, & Collin, 2018), *RHO* (Murray et al., 2015), and *USH2A* (Slijkerman et al., 2016), and represents a promising therapy for retinal dystrophies (Collin & Garanto, 2017).

In conclusion, we identified a pathogenic NEAR splice variant in *TULP1* in trans with a known pathogenic missense variant in two siblings with an early‐onset photoreceptor dystrophy in whom analysis of the exonic and consensus splice site regions did not identify the cause of disease. This highlights the importance of investigating noncoding regions in order to obtain a conclusive molecular diagnosis in patients with a hereditary retinal dystrophy.

## CONFLICT OF INTEREST

The authors have no proprietary or commercial interest in any materials discussed in the article.
